# Comparative Efficacy and Safety of Once-Weekly Semaglutide Formulations in Indian Adults With Obesity: A Phase III, Randomized Non-inferiority Active-Controlled Study (Size Plus Study)

**DOI:** 10.7759/cureus.105749

**Published:** 2026-03-24

**Authors:** Nitin Kapoor, Sanjay Kalra, Arindam Naskar, Shehla Shaikh, Sambit Das, Sunil Kota, Saptarshi Bhattacharya, Raja Bhattacharya, Richa Giri, Niteen Karnik, Gandhi Parise, Aruna Mangipudi, Amritava Ghosh, Amol Dange, Deepak Khandelwal, Mayura Chaudhari, Narayan Deogaonkar, Paramesh Shamanna, Sanket Sorate, Santosh Saklecha, Sheloje Samadhan, Deepak Varade, Jayashree Shembalkar, Kushal Bangar, Nilesh Lomte, Mayur Mayabhate, Nitin Kapure, Akhilesh Sharma, Radhakrishna Vaddem, Mukesh Jaiswal

**Affiliations:** 1 Department of Endocrinology, Christian Medical College and Hospital, Vellore, IND; 2 Department of Endocrinology, Bharti Hospital, Karnal, IND; 3 Department of Endocrinology, Nutrition and Metabolic Diseases, School of Tropical Medicine, Kolkata, IND; 4 Department of Endocrinology, Prince Aly Khan Hospital, Mumbai, IND; 5 Department of Endocrinology, Kalinga Institute of Medical Sciences, Bhubaneswar, IND; 6 Department of Endocrinology, Diabetes Clinic, Berhampur, IND; 7 Department of Endocrinology, Indraprastha Apollo Hospitals, New Delhi, IND; 8 Department of Medicine, Medical College and Hospital, Kolkata, IND; 9 Department of Medicine, Ganesh Shankar Vidyarthi Memorial (GSVM) Medical College, Kanpur, IND; 10 Department of Medicine, Lokmanya Tilak Municipal Medical College & General Hospital, Mumbai, IND; 11 Department of Medicine, Government Siddhartha Medical College, Vijayawada, IND; 12 Department of Endocrinology, King George Hospital, Vizag, IND; 13 Department of Endocrinology and Metabolism, All India Institute of Medical Sciences, Raipur, IND; 14 Department of Medicine, Lifepoint Multispecialty Hospital, Pune, IND; 15 Department of Endocrinology and Diabetes, Khandelwal Diabetes, Thyroid and Endocrinology Clinic, New Delhi, IND; 16 Department of Medicine, Ishwar Institute of Health Care, Chhatrapati Sambhajinagar, IND; 17 Department of Medicine, Deogaonkar Multispeciality Hospital, Nashik, IND; 18 Department of Diabetes and Endocrinology, Bangalore Diabetes Centre, Bangalore, IND; 19 Department of Medicine, Sanjeevani Criticare and Research Centre Pvt. Ltd., Nashik, IND; 20 Department of Medicine, Santosh Hospital, Bangalore, IND; 21 Department of Medicine, Medipoint Hospitals Pvt. Ltd., Pune, IND; 22 Department of Medicine, Ashirwad Hospital, Mumbai, IND; 23 Department of Endocrinology, Getwell Hospital and Research Institute, Nagpur, IND; 24 Department of Medicine, Asian Institute of Medical Sciences, Mumbai, IND; 25 Department of Medicine, Galaxy Superspeciality Hospital and Research Center, Chhatrapati Sambhajinagar, IND; 26 Medical Affairs, Alkem Laboratories Ltd., Mumbai, IND

**Keywords:** body mass index: bmi, glp-1 receptor agonists, non-inferiority study, semaglutide, weight loss and obesity

## Abstract

Introduction: Obesity is highly prevalent in India, creating an urgent need for effective management interventions. The study hypothesizes that synthetic semaglutide has comparable safety and efficacy to the innovator drug when used in obese adults for weight management.

Methods: A phase III multicenter randomized active-controlled non-inferiority trial enrolled adults with obesity across 19 centers in India. Subjects were randomized to the test arm receiving synthetic semaglutide (Alkem Laboratories Limited) or the reference arm administered with innovator semaglutide (Wegovy®, Novo Nordisk) over 24 weeks in a 2:1 ratio. The primary efficacy endpoint was the percentage change in body weight,24 weeks post-intervention. Synthetic semaglutide was established to be non-inferior if the lower bound of the one-sided 97.5% confidence interval for the between-group difference did not exceed 4.5%.

Results: Of the 249 randomized participants, 246 (98.8%) completed the study. Mean percentage weight loss after 24 weeks was -14.39 ± 4.17% in the test arm and -14.61 ± 4.36% in the reference arm. The least square-mean difference was 0.15% (-0.93 to 1.24), meeting the predefined non-inferiority criterion. Weight loss >10% was achieved by 86.67% (n=143) in the test arm and 83.95% (n=68) in the reference arm (p = 0.5666), while >15% weight loss occurred in 38.79% (n=64) and 40.74% (n=33), respectively (p = 0.7683). Mean body mass index decreased by -4.93 ± 1.43 kg/m² in the test arm and -5.00 ± 1.50 kg/m² in the reference arm (p = 0.7128). Treatment-emergent adverse events were reported in 55.42% (n=92) of test-arm participants and 54.22% (n=45) of reference-arm participants.

Conclusions: Test semaglutide demonstrated non-inferior efficacy, comparable safety, and similar tolerability to the innovator product.

## Introduction

The World Health Organization (WHO) reported that more than 890 million adults around the world are obese and that the number of obese adults has doubled since 1990 [1]. A prevalence rate of 40.3% was documented in a large-scale cross-sectional study including 1,00,531 Indians [2]. The increasing prevalence of obesity is a public health issue, linked to various cardiovascular risk factors, including dyslipidemia, hypertension, impaired glucose metabolism, systemic inflammation, and cardiovascular diseases [3]. A 5% loss of initial body weight may lower the risk of obesity-related health problems, and losing more weight may have even more health benefits [4]. Beyond physical well-being, obesity adversely affects psychological health, increasing vulnerability to poor self-esteem, negative body image, and mood disorders [5].

The cornerstone for weight management is lifestyle changes, including dietary adjustments and enhanced physical activity [6]. Decreasing calorie intake to 1200-1800 kcal per day or achieving ≥500 kcal energy deficit every day promotes weight reduction. Individuals are advised to undertake at least 150 minutes of moderate-intensity physical activity distributed over 3-5 sessions per week [6]. While lifestyle interventions are the most recommended approach for reducing weight, there are significant challenges like insufficient weight reduction or weight regain in the long-term [7]. Therefore, clinical guidelines advocate administration of adjunctive pharmacotherapy in adults with a body mass index (BMI) ≥30, or ≥27 with comorbidities present [8-10].

Antiobesity medications (AOMs) facilitate greater and sustained weight loss by controlling the dysregulated appetite mechanisms [11]. Several drugs are approved by the Food and Drug Administration (FDA) and the European Medicines Agency (EMA) for the management of obesity, including tirzepatide, semaglutide, liraglutide, orlistat, naltrexone/bupropion, and phentermine/topiramate [12]. Tirzepatide is currently the only agent approved as an AOM in India, with other therapies being used off-label or awaiting regulatory approval. Among the available pharmacological options, semaglutide, a glucagon-like peptide-1 receptor agonist (GLP-1RA), is a potent drug for weight loss, with established efficacy in landmark clinical trials [13,14].

Initially, semaglutide received regulatory approval for type 2 diabetes. In 2021, the FDA extended approval of subcutaneous semaglutide for chronic weight management at higher weekly doses of 1.7 and 2.4 mg [7]. A large-scale, randomized controlled trial (RCT) reported that semaglutide decreased the mean body weight by ~15%, compared with 2.4% with placebo after 68 weeks of treatment; 32% of the treated individuals achieved ≥20% weight loss versus 1.7% in the placebo group [14]. Greater weight reduction translates to improved cardiometabolic profile, including positive changes in glycemic indices, blood pressure, lipid parameters, and waist circumference [7,14].

While semaglutide can potentially improve obesity care, the high cost limits the accessibility and widespread utilization for weight management in India. In this context, we have developed a semaglutide formulation hypothesized to be comparable and non-inferior to the innovator injectable semaglutide. The primary objective of this study was to establish non-inferiority based on the percentage change in body weight at 24 weeks. Secondary objectives included assessing the proportion of participants achieving ≥10% and ≥15% weight loss, changes in BMI, waist circumference, and HbA1c, and safety and immunogenicity evaluations.

## Materials and methods

Trial design and oversight

A phase III, multicenter, randomized, parallel-group, active-controlled, non-inferiority study included 249 patients with obesity, across 19 centers in India, to compare the safety and efficacy of a test semaglutide with that of innovator semaglutide for weight reduction.

Written informed consent was collected from all participants at each participating site. The study was approved by the institutional ethics committee at each centre, and all procedures complied with the Declaration of Helsinki and applicable local regulations. The trial was recorded in the Clinical Trial Registry of India CTRI/2025/04/085455 on 23/04/2025.

Participants

Adult participants (>18 years) were included, presenting with obesity, defined as a BMI ≥ 30 kg/m² or a BMI ≥ 27 kg/m² with ≥ one weight-related comorbidity, and at least one prior unsuccessful attempt at weight loss through dietary intervention. A serum calcitonin concentration < 50 ng/L was required.

Key exclusion criteria included the presence of diabetes (HbA1c ≥ 6.5% or previously diagnosed diabetes), recent use of glucose-lowering therapies or GLP-1 RA, recent significant weight change (>5 kg within three months prior to screening), or use of pharmacologic or surgical obesity treatments. Participants with uncontrolled thyroid disease, significant psychiatric disorders (including recent major depressive disorder, severe psychiatric illness, or suicidal ideation or behavior), or a history of substance abuse were excluded. Patients with a history of pancreatitis, family history of multiple endocrine neoplasia type 2, and medullary thyroid carcinoma were also excluded.

Procedures

The participants were randomized to the test and reference arms in a 2:1 ratio. The test semaglutide (Alkem Laboratories Limited) was administered to the participants in the test arm, while the reference arm received innovator semaglutide (Inj. Wegovy® Novo Nordisk). Both treatments were administered once per week and followed a dose-escalation schedule as defined [13]. Participants initiated treatment at 0.25 mg/week for 1-4 weeks, 0.5 mg/week during 5-8 weeks, 1 mg/week during 9-12 weeks, 1.7 mg/week during 13-16 weeks, and 2.4 mg/week from week 17 through week 24. Subcutaneous injections were administered once every week, either in the abdomen, thigh, or upper arm. The treatment was strictly taken on the same day of the week for the complete study period. The injection could be administered at any time of the specified day, with or without food.

A standard counselling for diet and lifestyle modification was provided at week 0 and continuously reinforced throughout the study. Patients were counselled every four weeks to help them adhere to a reduced-calorie diet (500-kcal deficit per day relative to the energy expenditure) and increased physical activity (150 minutes per week of physical activity). Compliance to dietary recommendations and lifestyle modification was documented in the patient diary and monitored throughout the study period.

The Interactive Web Response System (IWRS) was utilized for centralized randomization. A unique number was randomly assigned to every study participant, with each number corresponding to a treatment group based on a predefined schedule. A single-blind design was employed, in which investigators were blinded to treatment allocation, while participants were not blinded to treatment allocation. At every investigational site, an unblinded study coordinator managed investigational product dispensing, provision of dosing instructions, administration training to participants, and maintained investigator blinding throughout the study period. Due to inherent differences between the test and reference products, participants were not blinded. To minimize bias, all investigators and site staff involved in efficacy and safety assessments were strictly blinded, and laboratory personnel and adjudicators had no access to randomization codes. The unblinded coordinator functioned independently to ensure maintenance of investigator blinding throughout the study, thereby preserving the integrity of both subjective and objective outcome assessments.

Endpoints and assessments

The primary endpoint of the trial was to assess the percentage body weight loss. Secondary endpoints included (i) the percentage of participants achieving >10% and >15% weight reduction, (ii) changes in BMI, waist circumference, and HbA1c, (iii) the rate of adverse events, and (iv) the percentage of participants developing anti-drug antibodies (ADAs).

At each visit, clinical assessments were documented, encompassing body weight, waist-to-hip ratio, waist circumference, and BMI. A physical exam included fundoscopy and electrocardiography (ECG). After resting for five minutes, vital signs were taken while lying down. Blood samples were collected to assess HbA1c and ADA levels. Adverse events were either reported by participants or observed by investigators and meticulously documented throughout the study period.

Statistical analyses

Statistical analyses were performed using R 4.3.0 (R Foundation for Statistical Computing, Vienna, Austria). Efficacy analyses were conducted in the per-protocol population, participants completing the study without major protocol deviations. Analysis of covariance (ANCOVA) models were used for continuous efficacy outcomes. For each analysis, the treatment group and the baseline BMI category (< 32.5 vs ≥ 32.5 kg/m²) were fixed effects, and the baseline value was used as a covariate. The ANCOVA model was used to derive the least square (LS) mean difference with 95% confidence interval (CI).

The test semaglutide was considered non-inferior to the reference innovator semaglutide if the lower limit of the one-sided 97.5% CI for the treatment difference in mean percentage change in body weight at Week 24 was below 4.5%. The non-inferiority margin of 4.5% was selected based on clinical relevance and regulatory principles. In the STEP 1 Trial [14], semaglutide 2.4 mg demonstrated a placebo-subtracted weight reduction of -12.4%. The chosen margin preserves more than one-third of this established effect, consistent with regulatory expectations for non-inferiority trials. Differences <4.5% are unlikely to yield clinically meaningful differences in cardiometabolic outcomes or quality of life when both arms receive identical GLP-1 therapy. As a ≥5% reduction in body weight is considered clinically meaningful, a 4.5% margin ensures a conservative and clinically justified threshold.

Paired t-tests or Wilcoxon signed-rank tests were used to assess changes within groups from the baseline. Categorical variables were reported as frequencies and percentages. Chi-square tests or Fisher’s exact tests were used for inter-group comparisons, as suitable. 

Safety analyses were performed in the intention-to-treat population, including data from all participants receiving at least one dose of study medication. Adverse events, laboratory parameters, vital signs, ECG findings, fundoscopy, and physical examination findings were included in safety data. Descriptive statistics were used to report continuous safety variables, and frequencies and percentages were used to report categorical variables. For all analyses, missing data were handled using the last-observation-carried-forward method.

Sample size calculation

Assuming a non-inferiority margin of 4.5%, an expected difference of 0 between treatments, and a standard deviation of 11, with a one-sided alpha of 2.5% and 80% power, the required sample size was estimated to be 210 participants (140 in the test arm and 70 in the reference arm). After accounting for 15% dropout rate, the total sample size was determined as 249 participants.

## Results

Study participants

**Figure 1:**
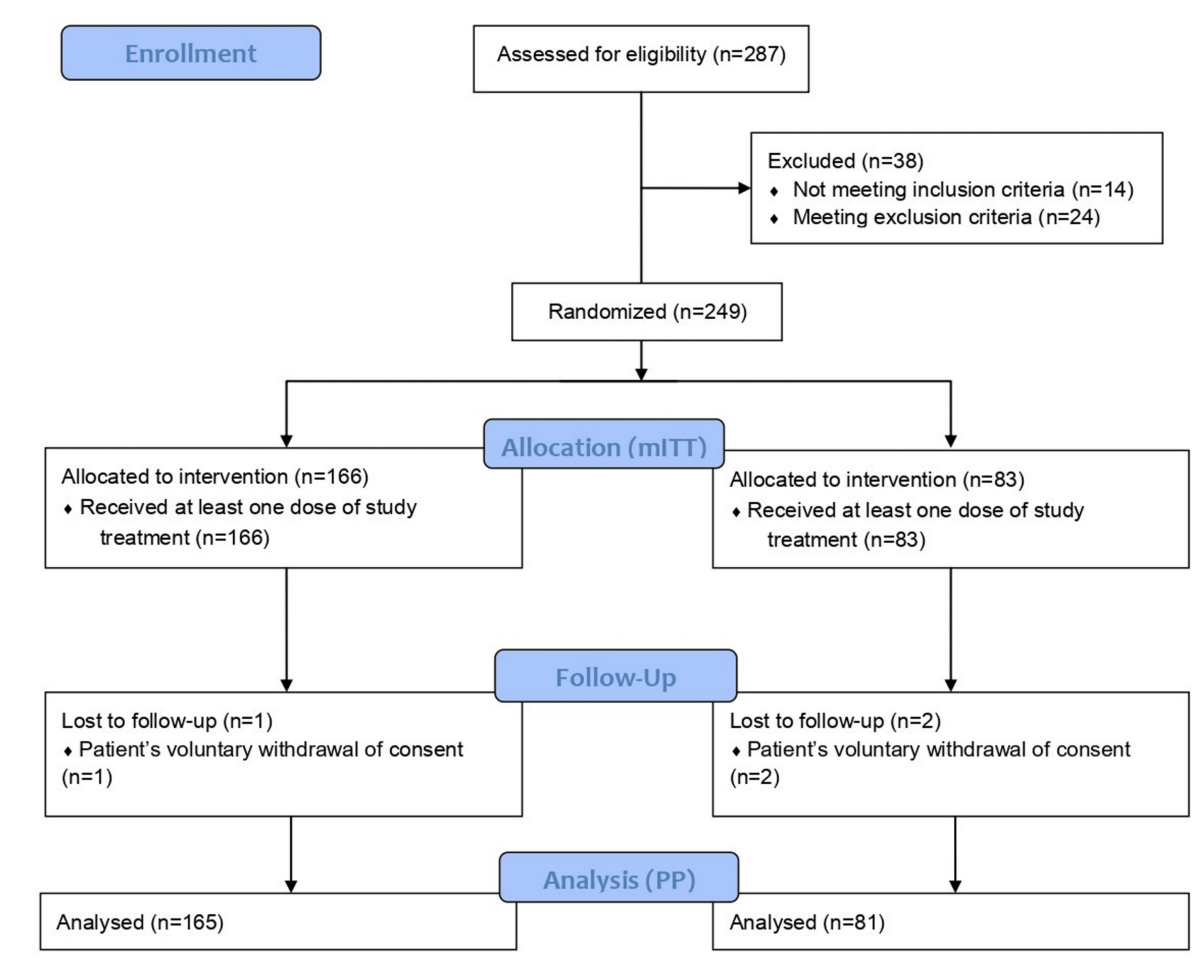
Consort diagram showing the flow of participants through the study mITT: Modified intent to treat population; PP: Per-protocol population

The study included 249 participants, 166 (66.67%) in the test arm and 83 (33.33%) in the reference arm (Table 1). Out of these, 246 subjects (98.80%) completed the study (test arm: n=165, 99.4%; reference arm: n=81, 97.59%) (Figure 1). Out of the total study subjects, 133 (53.41%) were male patients, and 116 (46.59%) were female patients. At baseline, the mean age of participants in the test arm was 39.42 ± 10.81 years and 38.65 ± 10.33 years in the reference arm, with no significant difference. The mean weight, BMI, waist circumference, and waist-to-hip ratio at baseline were comparable between the groups (p>0.05).

**Table 1:** Summary of patient demographic data (N=269) All values are represented as mean (SD). BMI: Body Mass Index; SD: Standard Deviation

Parameters	Test (N=166)	Reference (N=83)	Overall (N=249)	p-value
Age (years)	39.42 (10.81)	38.65 (10.33)	39.17 (10.63)	0.5901
Height (cms)	162.47 (8.40)	161.51 (7.02)	162.15 (7.96)	0.3705
Weight (kg)	91.72 (15.82)	90.64 (16.30)	91.36 (15.96)	0.6154
BMI (kg/m^2^)	34.71 (5.05)	34.66 (5.24)	34.70 (5.10)	0.9365
Waist circumference (inches)	41.80 (4.93)	42.38 (5.08)	41.99 (4.98)	0.3895
Waist-to-hip ratio	0.92 (0.07)	0.94 (0.06)	0.93 (0.06)	0.0599

Treatment effect on body weight

**Figure 2:**
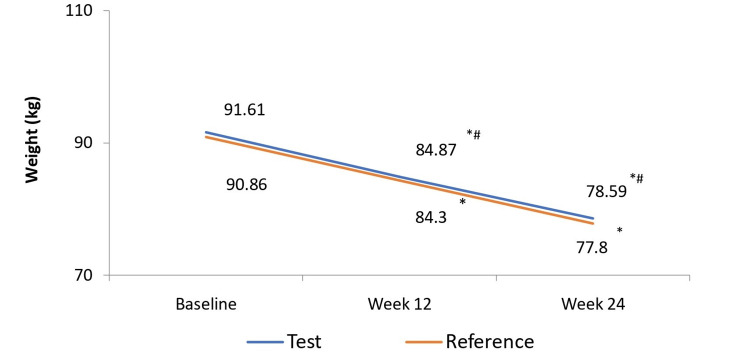
Change in mean body weight from baseline to week 24 in test (N=165) and reference (N=81) arms Values represent mean body weight (kg) at baseline, week 12, and week 24. Percent reduction in body weight from baseline to week 24 was significant in both arms (*p<0.0001 for both). Between-group comparison showed comparable weight reduction (# p = 0.7790).

After 24 weeks of treatment, the mean percent change in body weight from baseline was -14.39 ± 4.17% in the test arm and -14.61 ± 4.36% in the reference arm (p < 0.0001 in both arms). The between-group comparison indicated that the magnitude of weight reduction was comparable between both treatments and was lower than the predefined non-inferiority criterion of 4.5% (LS-mean difference: 0.15%; 95% CI: -0.93, 1.24; p = 0.7790) (Figure 2).

At week 24, 86.67% (n=143) of participants in the test arm and 83.95% (n=68) in the reference arm achieved >10% body weight loss (p = 0.5666). Furthermore, 38.79% (n=64) participants in the test arm and 40.74% (n=33) in the reference arm achieved >15% body weight loss (p = 0.7683). The between-treatment difference for both thresholds was not statistically significant, indicating comparable categorical weight-loss efficacy (Table 2).

**Table 2:** Proportion of patients who achieved>10% and >15% weight loss at 24 weeks (N=246)

Percentage weight loss	Test(N=165)	Reference (N=81)	Overall (N=246)	p-value
≤10%	22 (13.33%)	13 (16.05%)	35 (14.23%)	0.5666
>10%	143 (86.67%)	68 (83.95%)	211 (85.77%)	0.5666
≤15%	101 (61.21%)	48 (59.26%)	149 (60.57%)	0.7683
>15%	64 (38.79%)	33 (40.74%)	97 (39.43%)	0.7683

Treatment effect on BMI

**Figure 3:**
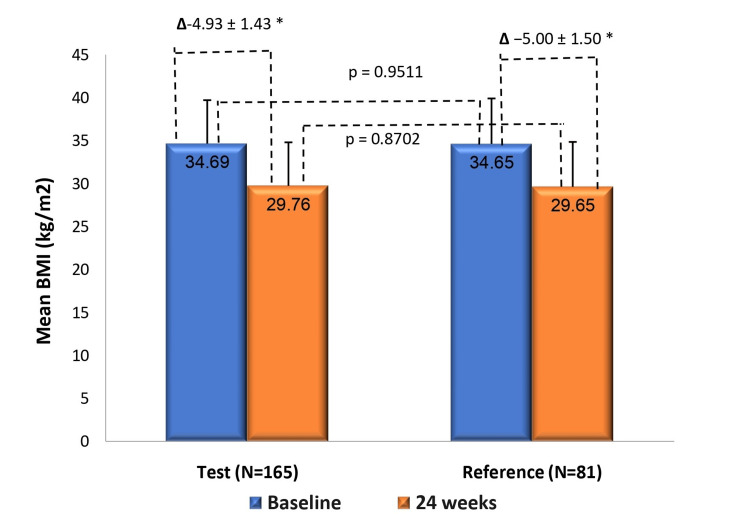
Change in mean BMI from baseline to week 24 in test (N=165) and reference (N=81) arms BMI reduction from baseline to week 24 was significant in both arms (*p<0.0001 for both). Between-group comparison showed comparable reductions.

The mean (± standard deviation (SD)) change in BMI was -4.93 ± 1.43 kg/m^2^ in the test arm and -5.00 ± 1.50 kg/m^2^ in the reference arm (p < 0.0001 for both) from baseline to 24 weeks. The between-group comparison demonstrated that the reductions were comparable (LS-mean difference: 0.07 kg/m^2^; 95% CI: -0.31, 0.46; p = 0.7128) (Figure 3).

Treatment effect on waist circumference

**Figure 4:**
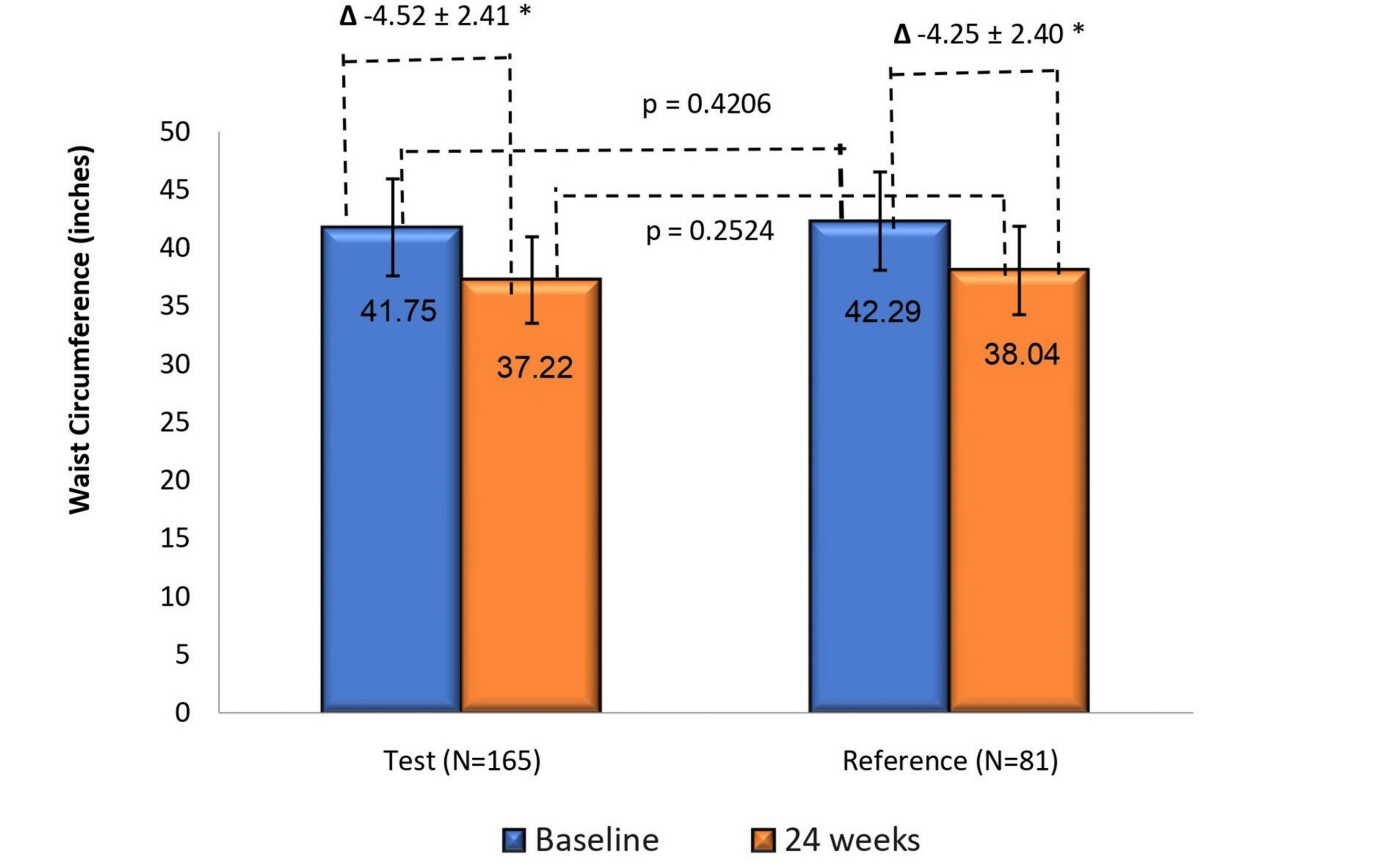
Change in mean waist circumference from baseline to week 24 in test (N=165) and reference (N=81) arms Waist circumference reduction from baseline to week 24 was significant in both arms (*p<0.0001 for both). Between-group comparison showed comparable reductions

Post-24 weeks of treatment, the mean (± SD) change in waist circumference from baseline was -4.52 ± 2.41 inches in the test arm and - 4.25 ± 2.40 inches in the reference arm (p < 0.0001 in both arms). The between-group reductions were comparable (LS-mean difference: -0.32 inch; 95% CI: -0.96, 0.32; p = 0.3209) (Figure 4).

Treatment effect on HbA1c

**Figure 5:**
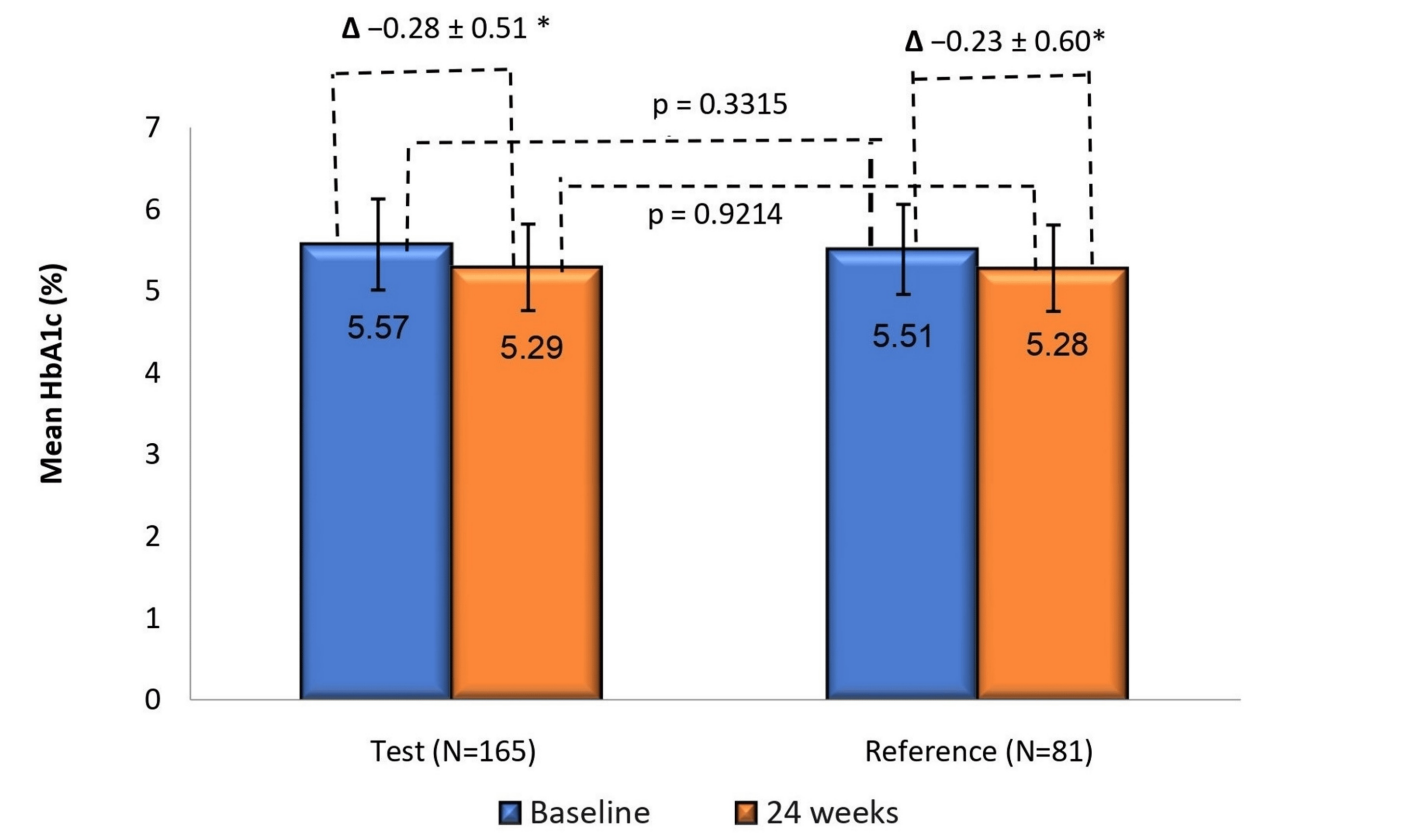
Change in mean HbA1c from baseline to week 24 in test (N=165) and reference (N=81) arms HbA1c reduction from baseline to week 24 was significant in the test arm (*p<0.0001) and reference arm (p = 0.0009). Between-group comparison showed comparable reductions

From baseline to week 24, the mean (± SD) change in HbA1c was -0.28 ± 0.51% in the test arm (p < 0.0001) and -0.23 ± 0.60% (p = 0.0009) in the reference arm. Between-group analysis showed an LS-mean change of -0.27% (95% CI: -0.33, - 0.20) in the test arm and -0.26% (95% CI: -0.35, -0.16) in the reference arm, with an LS-mean difference of -0.01% (95% CI: -0.12, 0.11; p = 0.8928) (Figure 5).

Safety outcomes

A total of 203 treatment-emergent adverse events (TEAEs) were reported in 92 subjects (55.42%) in the test arm and 104 TEAEs in 45 subjects (54.22%) in the reference arm (Table 3). Most adverse events had mild or moderate severity and were predominantly related to gastrointestinal disorders and general system disorders (Table 4). There was one serious adverse event (SAE) reported in the test arm (0.60%), and the event was related to the test treatment. No SAEs were reported in the reference arm.

**Table 3:** Summary of adverse events N: Number of subjects dosed with each treatment; n: Number of subjects with adverse event with particular category; %: Calculated using the number of subjects treated with each treatment as the denominator (n/N*100) AE: Adverse event; SAEs: Serious adverse events; TEAEs: Treatment-emergent adverse events

Category	Sub-Category	Number of Events			n (%)		
		Test (N=166)	Reference (N=83)	Overall (N=249)	Test (N=166)	Reference (N=83)	Overall (N=249)
All AE	-	203	104	307	92 (55.42%)	45 (54.22%)	137 (55.02%)
All TEAEs	-	203	104	307	92 (55.42%)	45 (54.22%)	137 (55.02%)
Severity of TEAEs	Grade 1	180	82	262	84 (50.60%)	41 (49.40%)	125 (50.20%)
-	Grade 2	22	22	44	17 (10.24%)	8 (9.64%)	25 (10.04%)
-	Grade 3	1	0	1	1 (0.60%)	0 (0.00%)	1 (0.40%)
Outcome	Ongoing (Not resolved or stabilized on follow-up)	1	0	1	1 (0.60%)	0 (0.00%)	1 (0.40%)
-	Recovered/resolved	202	104	306	91 (54.82%)	45 (54.22%)	136 (54.62%)
Relationship	Related	175	88	263	85 (51.20%)	42 (50.60%)	127 (51.00%)
-	Unrelated	28	16	44	19 (11.45%)	10 (12.05%)	29 (11.65%)
Action taken regarding study treatment	None	203	104	307	92 (55.42%)	45 (54.22%)	137 (55.02%)
SAEs	-	1	0	1	1 (0.60%)	0 (0.00%)	1 (0.40%)
Seriousness criteria of TEAEs	-	1	0	1	1 (0.60%)	0 (0.00%)	1 (0.40%)
Hospitalization Or Prolongation of Existing Hospitalization	-	1	0	1	1 (0.60%)	0 (0.00%)	1 (0.40%)
Causality to Investigational Product	Related	1	0	1	1 (0.60%)	0 (0.00%)	1 (0.40%)

**Table 4:** Incidence of TEAEs by system organ class (SOC) and preferred term in both treatment groups N: Number of subjects dosed with each treatment; n: Number of subjects with adverse event with particular category; %: calculated using the number of subjects treated with each treatment as the denominator (n/N*100); TEAEs: Treatment-emergent adverse events

System Organ Class	Preferred Term	Number of Events			n (%)		
		Test (N=166)	Reference (N=83)	Overall (N=249)	Test (N=166)	Reference (N=83)	Overall (N=249)
Blood and lymphatic system disorders	-	1	0	1	1 (0.60%)	0 (0.00%)	1 (0.40%)
-	Anaemia	1	0	1	1 (0.60%)	0 (0.00%)	1 (0.40%)
Gastrointestinal disorders	-	150	76	226	77 (46.39%)	37 (44.58%)	114 (45.78%)
-	Abdominal distension	2	1	3	2 (1.20%)	1 (1.20%)	3 (1.20%)
-	Abdominal pain	3	3	6	3 (1.81%)	3 (3.61%)	6 (2.41%)
-	Abdominal pain upper	4	0	4	3 (1.81%)	0 (0.00%)	3 (1.20%)
-	Constipation	8	0	8	7 (4.22%)	0 (0.00%)	7 (2.81%)
-	Diarrhoea	31	21	52	25 (15.06%)	16 (19.28%)	41 (16.47%)
-	Gastritis	25	21	46	21 (12.65%)	13 (15.66%)	34 (13.65%)
-	Gastrooesophageal reflux disease	2	2	4	2 (1.20%)	1 (1.20%)	3 (1.20%)
-	Haemorrhoidal haemorrhage	1	0	1	1 (0.60%)	0 (0.00%)	1 (0.40%)
-	Hyperchlorhydria	15	8	23	11 (6.63%)	8 (9.64%)	19 (7.63%)
-	Nausea	14	5	19	10 (6.02%)	3 (3.61%)	13 (5.22%)
-	Vomiting	45	15	60	32 (19.28%)	13 (15.66%)	45 (18.07%)
General disorders and administration site conditions	-	17	11	28	15 (9.04%)	9 (10.84%)	24 (9.64%)
-	Asthenia	6	2	8	5 (3.01%)	2 (2.41%)	7 (2.81%)
-	Chest pain	1	0	1	1 (0.60%)	0 (0.00%)	1 (0.40%)
-	Pain	0	1	1	0 (0.00%)	1 (1.20%)	1 (0.40%)
-	Pyrexia	10	8	18	10 (6.02%)	7 (8.43%)	17 (6.83%)
Infections and infestations	-	3	1	4	3 (1.81%)	1 (1.20%)	4 (1.61%)
-	Gastroenteritis	1	0	1	1 (0.60%)	0 (0.00%)	1 (0.40%)
-	Nasopharyngitis	2	1	3	2 (1.20%)	1 (1.20%)	3 (1.20%)
Injury, poisoning and procedural complications	-	1	0	1	1 (0.60%)	0 (0.00%)	1 (0.40%)
-	Contusion	1	0	1	1 (0.60%)	0 (0.00%)	1 (0.40%)
Metabolism and nutrition disorders	-	23	11	34	14 (8.43%)	9 (10.84%)	23 (9.24%)
-	Decreased appetite	23	11	34	14 (8.43%)	9 (10.84%)	23 (9.24%)
Nervous system disorders	-	6	3	9	4 (2.41%)	3 (3.61%)	7 (2.81%)
-	Dizziness	0	1	1	0 (0.00%)	1 (1.20%)	1 (0.40%)
-	Headache	6	1	7	4 (2.41%)	1 (1.20%)	5 (2.01%)
-	Paraesthesia	0	1	1	0 (0.00%)	1 (1.20%)	1 (0.40%)
Respiratory, thoracic and mediastinal disorders	-	2	2	4	2 (1.20%)	2 (2.41%)	4 (1.61%)
-	Cough	2	2	4	2 (1.20%)	2 (2.41%)	4 (1.61%)

The incidence of ADA formation post administration was 4.85% (n=8) in the test arm and 3.7% (n=3) in the reference arm (p=0.6831). No treatment-emergent immunogenicity signal or increasing trend was observed. All post-dose ADA-positive subjects did not exhibit any hypersensitivity reactions, loss of efficacy, or ADA-related safety events. The findings indicate that immunogenicity following treatment was low and comparable between the test and reference semaglutide, with no associated hypersensitivity, safety concerns, or impact on efficacy.

## Discussion

This phase III trial demonstrated the non-inferiority of test semaglutide versus the innovator drug. Both interventions resulted in statistically significant body weight reductions of −14.39% and −14.61%, respectively, at 24 weeks, with no significant between-group difference (p = 0.7790). Categorical outcomes were comparable; >10% weight loss was achieved by 86.67% vs. 83.95% of participants (p = 0.5666), and >15% weight loss by 38.79% vs. 40.74% (p = 0.7683) in the test and reference arms, respectively. Safety profiles were similar, with TEAEs occurring in 55.42% of participants in the test arm and 54.22% in the reference arm. Overall, the test semaglutide demonstrated equivalent efficacy and safety to the innovator drug.

Treatment with the test semaglutide reduced absolute weight by 13.02 kg (−14.39%) after 24 weeks, consistent with the treatment effect size reported for the innovator drug. A meta-analysis of innovator semaglutide trials reported a weighted mean relative weight reduction of 12.1% and an absolute reduction of 12.3 kg with long-term treatment [15]. The STEP-1 trial reported 14.9% mean weight loss and an absolute reduction of 15.3 kg at 68 weeks [14], while the SELECT trial reported sustained reductions of 10.2% over 208 weeks [13]. Weight loss with semaglutide is mediated by appetite suppression through direct and indirect central nervous system mechanisms, reducing caloric intake [16-19]. These findings confirm that test semaglutide achieves weight-loss efficacy benchmarks set by the innovator drug.

At 24 weeks, 86.7% and 38.8% of patients receiving test semaglutide achieved >10% and >15% body weight loss, respectively; these proportions were comparable to the reference arm. By comparison, the STEP-1 trial reported 69.1% and 50.5% achieving ≥10% and ≥15% weight loss at 68 weeks [14], while a long-term study found 44.2% and 22.9% achieving these thresholds after 104 weeks [13]. In the present study, these targets were reached within a shorter timeframe; however, sustained efficacy over longer periods warrants further investigation.

Weight loss of 10-15% or greater is recommended for individuals with obesity-related complications such as prediabetes, hypertension, and obstructive sleep apnea [10,20-22]. A 5% weight loss improves hyperglycemia and blood pressure control [10], while 5-10% weight reduction may delay or prevent diabetes, polycystic ovary syndrome, liver disease, and dyslipidemia [10]. Losses of 10-15% reduce cardiovascular risk, urinary incontinence, steatohepatitis, sleep apnea, gastric reflux, and knee osteoarthritis [23], and losses exceeding 15% can achieve diabetes remission and reduce the risk of cardiovascular mortality and heart failure [24]. Achieving these targets is therefore a key therapeutic goal in obesity management.

The test drug was well-tolerated with no new or unexpected safety signals and was consistent with the established tolerability profile of semaglutide [13-15] and the GLP-1RA class [25]. TEAEs were reported in 55.42% of test-arm participants, predominantly presenting with gastrointestinal events, which was an expected class effect. A prior meta-analysis reported a higher incidence of 91.8% with long-term innovator semaglutide use [15]. Only one SAE (0.60%) occurred in the test arm, where the participant developed gastroenteritis (nausea, abdominal discomfort, diarrhea) on semaglutide 1 mg/week, requiring two-day hospitalization. The event resolved with supportive therapy and was assessed as possibly related to semaglutide. Long-term safety studies are needed to identify rare adverse events.

Apart from the efficacy and safety, affordability is a critical determinant of therapeutic uptake in developing economies such as India. Innovator semaglutide remains unaffordable to a large proportion of patients who could benefit from it [26]. The test semaglutide formulation evaluated here may offer comparable weight loss at a lower cost, improving accessibility and scalability. Formal cost-analysis studies are warranted to confirm cost-efficiency. Additionally, the unique obesity phenotype of Indians, characterized by higher visceral adiposity and lower BMI thresholds for diabetes onset [26], underscores the relevance of this study and supports semaglutide's integration into India's national obesity management frameworks and clinical practice recommendations.

Implications for obesity management

These findings support semaglutide's application in obesity management for appropriate patient populations. It serves as a valuable adjunct to behavioral therapy when lifestyle modification alone is insufficient. The clinically significant weight loss observed may confer durable metabolic benefits. The test formulation's comparable efficacy and tolerability suggest potential for interchangeable use in clinical practice and improved access to AOMs in cost-constrained settings of India. Ultimately, the effective and safe administration of semaglutide necessitates proper dose titration, patient education, and clinical oversight.

Strengths and limitations

The randomized controlled and multicenter design supports strong internal validity, providing clinically meaningful data on a potentially more affordable semaglutide formulation. High study completion and low TEAE-related dropouts minimize attrition bias. Inclusion of all randomized participants in the safety analysis ensures comprehensive adverse event assessment. 

Limitations include the short follow-up period of 24 weeks. As weight loss associated with GLP-1RAs continues beyond the first 24 weeks of treatment, the present study may not fully capture the maximal weight-loss effect or rare and long-term adverse effects. In addition, longer-term outcomes, including weight-loss maintenance, durability of response, and cardiometabolic benefits, cannot be determined within the timeframe of this study. In addition, the study only included people who actively consented to participate, which may represent a higher motivation toward lifestyle changes. Therefore, the study provides valuable insight into the efficacy of intervention under high adherence. Lastly, participants were not blinded to treatment, which may have introduced expectation bias, differential behavior, and reporting bias. However, this risk of performance and assessment bias was minimized by maintaining strict blinding of investigators and outcome assessors.

## Conclusions

This phase III non-inferiority trial exhibited similar efficacy, safety, and tolerability of the test semaglutide formulation compared to the innovator semaglutide, 24 weeks post-intervention in Indian adults with obesity. While longer-term studies are warranted to evaluate the durability of weight loss, cardiometabolic outcomes, and real-world effectiveness, the present findings support test semaglutide as a valuable adjunct to lifestyle modification for the management of obesity in clinical practice. Additionally, the test semaglutide formulation may increase the uptake of evidence-based pharmacotherapy for obesity in various healthcare contexts by possibly making it more affordable and increasing access in comparison to current branded formulations.
